# Sulphate Resistance of Alkali-Activated Material Produced Using Wood Ash

**DOI:** 10.3390/ma18184313

**Published:** 2025-09-15

**Authors:** Yiying Du, Ina Pundiene, Jolanta Pranckeviciene, Aleksandrs Korjakins

**Affiliations:** 1 Laboratory of Concrete Technology, Institute of Building Materials, Vilnius Gediminas Technical University, Linkmenų Str. 28, LT-08217 Vilnius, Lithuania; yiying.du@vilniustech.lt (Y.D.);; 2 Institute of Sustainable Building Materials and Engineering Systems, Faculty of Civil and Mechanical Engineering, Riga Technical University, Kipsalas St. 6A, LV-1048 Riga, Latvia; 3 Institute of Biomaterials and Bioengineering, Faculty of Natural Sciences and Technology, Riga Technical University, Paula Valdena Street 3, k-1, LV-1048 Riga, Latvia

**Keywords:** durability, waste valorisation, microstructure, aluminosilicates, phosphogypsum

## Abstract

The durability of construction and building materials under sulphate environments is an important indicator to evaluate their service life. In this study, the physical and mechanical behaviours of wood-ash-based alkali-activated materials (AAMs) incorporating coal fly ash, metakaolin, natural zeolite, and calcined phosphogypsum were assessed before and after being subjected to sodium sulphate corrosion cycles via the compressive strength, mass, and volume changes. The microstructure, elemental composition, and phase identification were further analysed using X-Ray Diffraction(XRD) and scanning electron microscope(SEM). The results show that the exposure to sulphate solution caused decalcification and dealumination of hydrates, releasing calcium and aluminium to react with sulphate and forming expansive erosion products, ettringite and gypsum. This contributed to the microstructural damage, leading to mass change, volume expansion, and compressive strength loss of 7.33, 1.29, and 60.42%. The introduction of binary aluminosilicate precursors enhanced the sulphate resistance by forming a well-bonded microstructure consisting of calcium (aluminate) silicate hydrate and sodium aluminate silicate hydrate, with the compressive strength loss decreasing up to 18.60%. The co-usage of calcined phosphogypsum deteriorated the mechanical properties of AAMs but significantly improved the sulphate resistance. The sodium sulphate environment facilitated anhydrate hydration, generating more sulphate hydrates and hemigypsums that co-existed with erosion products, forming a compact microstructure and improving the compressive strength by twofold.

## 1. Introduction

The worldwide construction industry in the modern context poses a significant demand for concrete manufacturing, which raises concerns about environmental burdens, particularly in relation to global warming. Nearly 4.4 billion tons of concrete materials are fabricated annually around the globe [1]. As its major binder material, ordinary Portland cement (OPC) is responsible for an estimated 5–7% of the entire anthropogenic emissions of greenhouse gases and takes up 12–15% of the total industrial energy consumption [2,3,4]. Under the background of sustainable development, the investigation of alternative cementless eco-binder materials has garnered increasing attention, including alkali-activated materials (AAMs) and geopolymers. In contrast to OPC, the AAM binder is estimated to reduce greenhouse gas emissions by up to 80% [5], significantly improving the sustainability of the concrete. In addition, the applications of industrial and agricultural wastes in AAMs exhibit great potential as an alternative approach for waste management instead of landfills [6,7,8,9,10], as well as construction and demolition wastes [11,12,13,14].

Wood ash (WA) is an industrial by-product generated during the combustion of wood chips and sawdust. It contains organic and inorganic contents that belong to the burnt wood residues. Around 6–10% of the wood mass transforms to WA during its combustion [15,16]. Annually, an average of 20 million tons of WA is produced worldwide, of which approximately 70% is disposed of in landfills [17]. This exerts significant burdens on the environment, especially with the growing application of biofuel energy as a renewable and clean energy source. Due to the diversity in WA characteristics, which heavily rely on the biomass categories and combustion technology, as well as its low chemical reactivity, the valorisation and reuse of WA remains a challenge. Some investigations have been conducted focusing on the incorporation of WA as a material to partially replace OPC or other binder materials [18,19,20,21]. However, it has been pointed out that WA can deteriorate the mechanical properties and rheological behaviours of OPC-based concrete due to its organic contents, particularly when a large amount of WA is added [15,22,23]. In addition, Zhu et al. [21] discovered that WA could worsen the compressive strength of phosphogypsum (PG)-based binder. When its substitution ratio grew to 50%, the strength decreased by 72.22%.

In contrast, positive effects of WA are reported when WA is incorporated in AAMs as a binary or ternary precursor source. For instance, Zhu et al. [21] provided insight into milled WA activated by sodium carbonate and sodium silicate as a green binder material, reporting that the partial substitution by phosphogypsum (PG) as a calcium source remarkably improved the mechanical properties of the binder. Vaiciukyniene et al. [24,25,26] applied WA as a binary precursor material together with PG, silica by-product blends, and zeolitic waste to fabricate alkali-activated composites, reaching the highest compressive strength of 30.0, 21.6, and 14.7 MPa, respectively. Tran Thi et al. [10] and Gomez-Casero et al. [27] used WA as a partial replacement of steel slag and slag-fly ash, respectively, highlighting that the addition of WA due to its filling effects can improve the mechanical properties and densify the microstructure. Other investigations also concern the utilisation of WBA with fly ash, metakaolin, and glass powder [28,29,30,31]. However, these studies show that when the content of WA reached over 30%, the strength showed a declining tendency, due to the low chemical reactivity of the ash. Considering the superior of its sustainability, it is thus necessary to investigate the possibility of the utilisation of WA at large quantities in producing AAMs.

Compared to the investigations on the enhancement of the mechanical properties of AAMs, fewer studies focus on durability assessment; however, those that do show outstanding durability performance of AAMs, in contrast with traditional OPC-based materials, such as great acid and alkali resistance and good freeze–thaw resistance [32,33]. Meanwhile, due to the special gel composition of AAMs, they generally have higher resistance against chloride diffusion and a retarded penetration by the chloride [33,34,35]. Water absorption and penetration are the mostly involved durability in the investigations, which is primarily influenced by the precursor types, activators, and testing methods [35]. In terms of the durability analysis of WA-based AAMs, currently, attention is only paid to water absorption tests [5], and studies concerning other types of durability tests remain rare. Due to the low specific surface area of WA, WA-based AAMs usually have a lower water absorption than those based on other types of precursor materials [5]. Sulphate attack is another common durability problem of concrete structures based on the source of the sulphate environment; it can be divided into external and internal attacks [36]. The former relates to sulphate exposure due to surrounding factors, such as sea water, soil contamination, decomposing organic matter, and industrial effluent [37,38], while the latter is caused by sulphate ions in the binder [39]. In both cases, sulphate ions permeate into the concrete and react with calcium- and aluminate-containing phases, generating erosion products, such as ettringite and gypsum [40,41], which account for expansion and deterioration of the materials. Traditional OPC-based concrete deteriorates severely during sulphate corrosion owing to the decomposition of the hydrates to form erosion products [42,43,44,45]. The sulphate attack on geopolymer materials is mainly affected by the categories of alkaline activators, the cations in the sulphate, and the water/binder ratio of the material [46,47]. Strength loss, mass change, volume change, and microstructural properties are the primary factors to evaluate the sulphate resistance of the geopolymers [48,49]. Similar to the erosion mechanism of OPC-based materials, for the high-Ca AAM system, the generation of expansive products due to the reaction of Ca-rich phases and sulphate solution is the primary cause of material deterioration [50,51]. In terms of low-Ca AAMs, the ion exchange between sulphate solutions and the internal matrix results in less swollen products, enhancing the resistance to sulphate corrosion [52]. Currently, a growing number of investigations are conducted on the resistance properties of AAMs against acidic environments, including sulphate erosion. However, most of these existing studies mainly focus on the AAMs based on coal fly ash (CFA), slag, and metakaolin (MK) as precursors [42,47,49,52,53,54]. Compared to OPC-based materials, the formation of geopolymeric gels due to the reaction of aluminosilicates in the precursor materials and alkaline solution, which shows less decomposition during sulphate attacks, reduces the production of expansive products and hence improves sulphate resistance. Regarding the sulphate resistance of WA-based AAMs, there are still no available studies, especially when exposed to extreme attack cycles.

To fill this gap, this research focused on the utilisation of a large amount of WA as precursors and studied the effects of sulphate attack cycles under extreme conditions on the produced AAMs, which broadens the knowledge of WA application in building and construction materials. Generally, sodium sulphate (Na_2_SO_4_) or magnesium sulphate (MgSO_4_) is used to prepare the sulphate environment. Considering that during the MgSO_4_ exposure, magnesium hydroxide (Mg(OH)_2_) can generate a protective layer on the specimen surface, reducing the erosion process [55], Na_2_SO_4_ was used as the sulphate source to create more extreme conditions. The aim of the study was to comprehensively evaluate the property changes of the WA-based AAM subjected to the extreme ambient conditions with sulphate solutions. Due to the consideration of improving the properties of WA-based AAMs, aluminosilicate-rich sources, including CFA, MK, and natural zeolite (NZ), were used as binary precursor materials to improve the contents of aluminate and silicate in the binder system. As a contrast, calcium-rich source binary precursor material, i.e., calcined phosphogypsum (CPG), was introduced as extra calcium contents to compare its impacts with aluminosilicates. These binary precursors were used at 20% of the total precursor mass. Their effects on sulphate resistance, as well as mechanical property development and microstructure, were analysed via physical characteristics, compressive strength, scanning electron (SEM) microscopy graphs, and X-ray diffraction (XRD) patterns.

## 2. Materials and Methods

### 2.1. Raw Materials

Precursor materials used in this study include a mixture of WA composed of 50% wood fly ash (WFA) and 50% wood bottom ash (WBA), coal fly ash (CFA), metakaolin (MK), natural zeolite (NZ), and calcined phosphogypsum (CPG). Natural river sand (NRS) was substituted by recycled sand (RS) as a fine aggregate due to considerations of improving eco-efficiency and sustainability. Both WA and RS were valorised from the wood combustion wastes. The source of the biomass was pine sawdust and chips, and the burning temperature was around 500–600 °C. RS came from NRS burnt together with biomass during the combustion process. Low-Ca CFA (Class F) was a by-product collected from a coal-burning power plant in Krakow, Poland. MK was a commercial product fabricated by Bauchemie GmbH (Bottrop, Germany). NZ was obtained from Ukraine. Phosphogypsum (PG) waste was recycled from the landfill of Lifosa Factory in Kedainiai, Lithuania.

**Figure 1:**
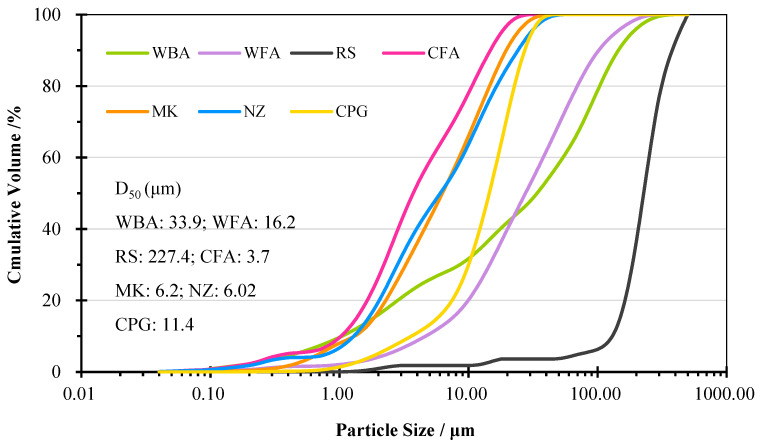
Particle size distribution of raw materials.

**Figure 2:**
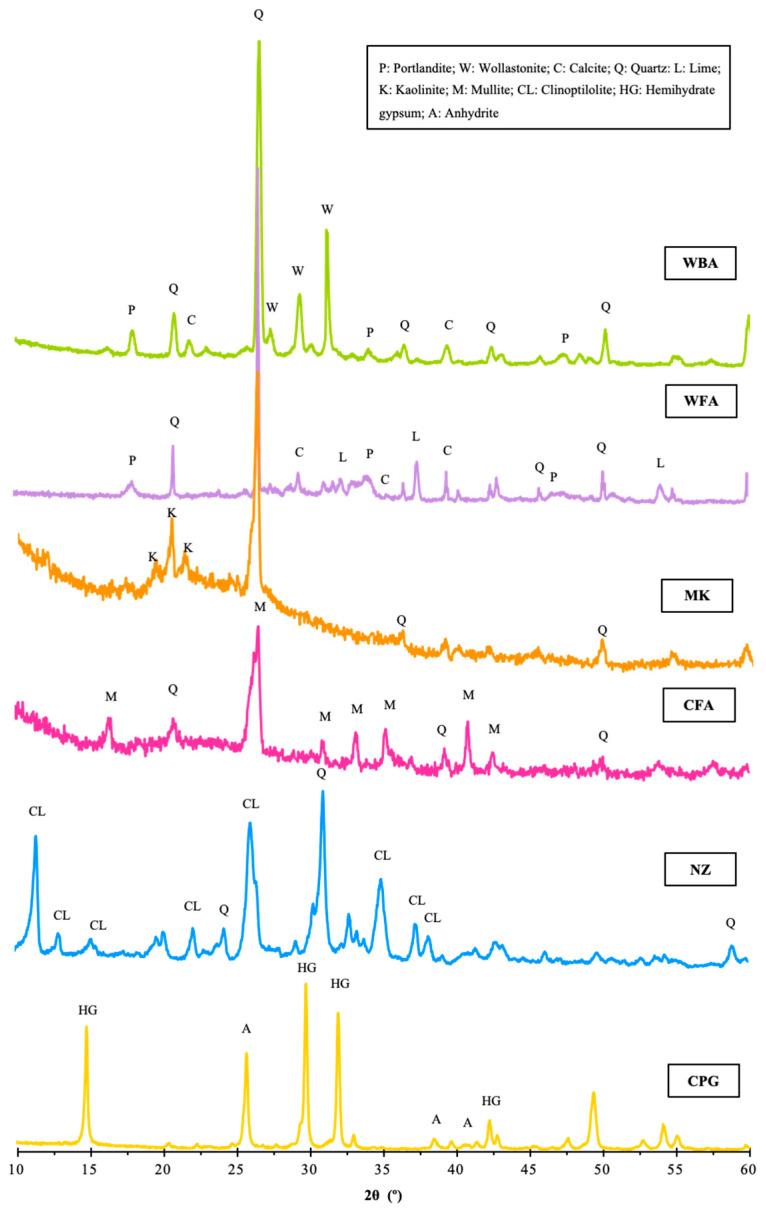
Mineralogical composition of raw materials.

Prior to the preparation of AAM, WA and CFA were ground using ball milling equipment for 24 h to decrease the particle size and improve their reactivity. RS was sieved through a 1.25 mm sieve. PG rocks were calcined in an oven at 200 °C for 24 h to produce calcined phosphogypsum (CPG), then it was milled for 48 h into a fine powder and sieved through a 0.5 mm sieve. The process of calcination transformed the calcium sulphate dihydrate (CaSO_4_·2H_2_O) into calcium sulphate hemihydrate (CaSO_4_·0.5H_2_O) [20]. The bulk density of the precursor materials was 1.03 (WBA), 0.57 (WF), 0.79 (CFA), 0.43 (MK), 0.24 (NZ), and 0.72 (CPG). The particle size distribution of the pre-treated raw materials is depicted in Figure 1. The particles of both WAs were mainly distributed between 1 and 100 μm, while those of other precursor materials varied between 1 and 10 μm, and RS had a particle size distribution from 100 to 1000 μm. The chemical compositions of the raw materials are depicted in Table 1 based on wavelength-dispersive X-ray fluorescence (XRF) analysis. Loss of ignition (LOI) was determined at 800 °C. WA and CPG were Ca-rich materials, while NZ, MK, and CFA were abundant in aluminosilicates. Figure 2 shows the mineralogical composition of the materials via X-ray diffraction (XRD) analysis. CPG was composed of hemihydrate gypsum and anhydrate. Besides quartz, in other raw materials, NZ, MK, and CFA also contained clinoptilolite, kaolinite, and mullite, respectively. In WAs, there was portlandite, lime, calcite, and wollastonite.

Alkaline activators are sodium hydroxide (SH), water glass (WG), and calcium hydroxide (CH). WG consisted of 50% water and 50% sodium silicate (SS). Its density was 1388 kg/m^3^. Boric acid (BA) was used as a retarder to decrease the initial setting time and ensure the workability of the AAM. Sodium sulphate decahydrate (Na_2_SO_4_·10H_2_O) was applied to produce sulphate solutions. All the chemical products were manufactured by Lerochem (Klaipeda, Lithuania).

### 2.2. Mix Proportions

Table 2 depicts the mix design, and the mix ratio by percentage is presented in Table 3. The water/precursor ratio was defined as 55%, and the precursor/fine aggregate ratio was 0.5. Due to the consideration to decrease the alkalinity in the system, the concentration of the SH solution was reduced to 7 mol/L in this study, and the WG was added, maintaining a molar ratio of SH/SS of 3. CH and BA were used at amounts of 10% and 1% by precursor mass, respectively. CH acted as a ternary alkaline activator and an extra source of calcium. The ratio of the CH was selected based on the previous investigation [56]. CFA, MK, NZ, and CPG replaced WA at 20% by total precursor mass as binary precursor materials. The proportion of the binary precursors was decided based on the preliminary tests. BA was added as a retarder to guarantee the workability. The initial setting time of the produced materials varied between 10 and 15 min, and the flowability ranged from 10 to 20 cm.

### 2.3. Preparation of Samples and Testing Procedures

Initially, the alkaline solution was prepared in advance by mixing the SH solution and WG. The dry mixture containing WA, RS, BA, CH, and binary precursors was stirred evenly for 5 min before the addition of the activator solutions. Then, the wet mixture was homogeneously stirred for 3 min in an electronic mixer. Afterwards, it was poured into steel prism moulds (160 mm × 40 mm × 40 mm) and subjected to a vibration table for 2 min to ensure compactness. The moulds were wrapped with plastic film and cured in a chamber with an elevated temperature of 60 °C. After 24 h, the prepared samples were demoulded and cured at room temperature for 28 days.

The chemical contents of the raw materials were obtained via an XRF spectrometer (AXIOS-MAX, Panalytical, Eindhoven, The Netherlands). The compressive strength before and after the sulphate attacks was examined using a WDW-20 universal testing apparatus. The XRD patterns of the raw materials and AAM samples were tested on an X-ray diffractometer from SmartLab (Rigaku, Tokyo, Japan) within a 2θ extent from 10 to 60°, and identification of the phases was completed using Highscore 3.0 software (PANalytical B.V., Almelo, The Neitherlands). The diffractometer is equipped with an X-ray tube of a 9 kW rotating Cu anode. The measurements were conducted based on the Bragg–Brentano principle with a graphite monochromator on the diffracted beam. The step scan mode has a step size of 0.02° (in 2θ scale) and a counting time of 1 s per step. Before being subjected to XRD and XRF, the samples were milled and sieved through a 0.063 mm sieve. Scanning electron microscopy and energy-dispersive X-ray spectroscopy (SEM-EDS) were conducted using a Thermalscientific Verios 5 UC with Silicon Drift Detector Oxford Instrument X-MaxN 150 equipment (Oxford, Britain). Before the test, the samples were crushed into small pieces, and the surfaces were polished.

**Figure 3:**
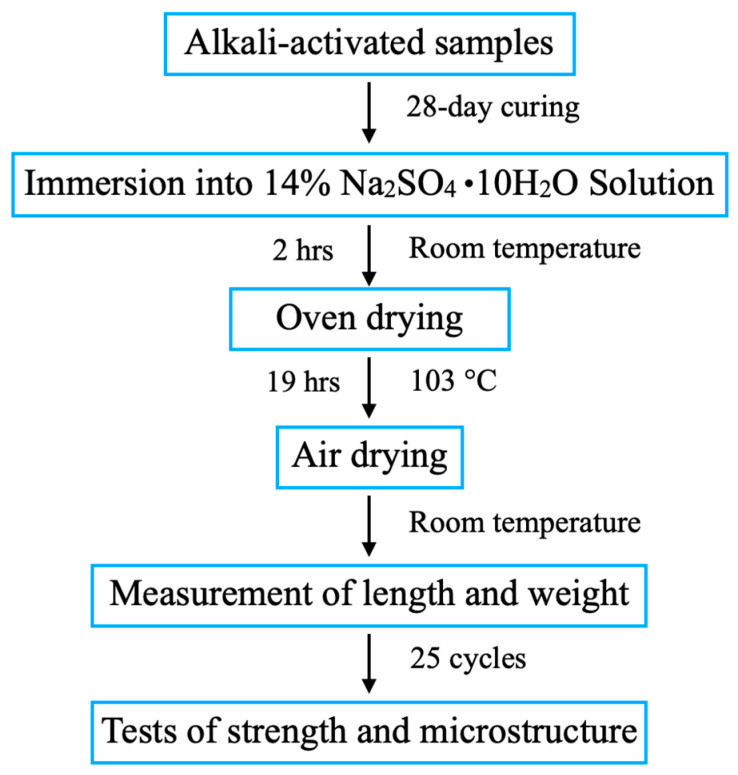
Research flow chart of sulphate attack.

As shown in Figure 3, the sulphate resistance of the AAM was assessed through corrosion cycles according to the standard EN 12370:2000. One cycle lasted for 24 h, and during each cycle, the samples were immersed in a 14% sulphate solution for 2 h at room temperature. Then, they were dried in an oven for 19 h at 103 °C. The measurement of the changes in mass and length was performed at the end of each cycle after air-drying the samples at room temperature. In the context of this study, 25 cycles were conducted, and afterwards, the changes in compressive strength and microstructure were measured. The compressive strength loss coefficient K was calculated as follows [36].

(1) $$K=\frac{f_{ct}}{f_{c0}}\times 100\%$$

where f_ct_ is the compressive strength after 25 sulphate attack cycles, and f_c0_ is the initial compressive strength.

## 3. Results

### 3.1. Mechanical Properties

**Figure 4:**
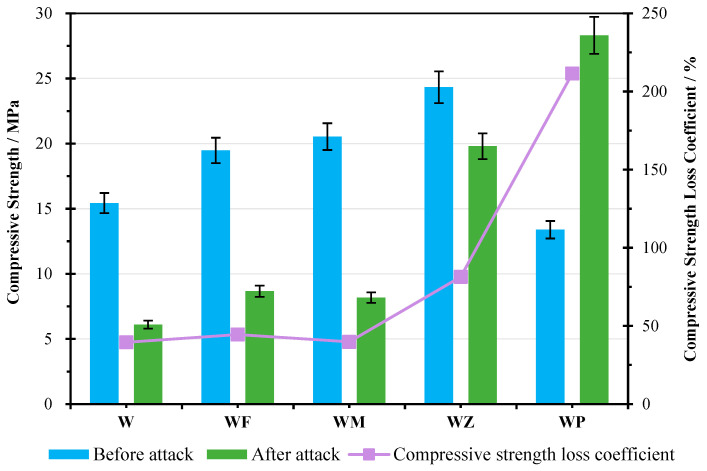
Comparison of compressive strength before and after sulphate attack.

The compressive strength of the produced AAM before and after the sulphate attack is presented, together with the compressive strength loss coefficient K, in which a percentage over 100% indicates the growth of the strength after immersion (Figure 4). The strength changing data are summarised in Table 4. Before being subjected to the sulphate solution, the specimen W with only WA had a compressive strength of 15.44 MPa. The utilisation of a secondary aluminosilicate precursor material (CFA, MK, and NZ) at 20% greatly improved the strength by 26.15, 33.02, and 57.58%, respectively, compared to W. The reason behind this could be that the increment of the active aluminates and silicates facilitated the hydration process to produce more calcium aluminate silicate hydrates (CASHs) and calcium silicate hydrates (CSHs). This was also discussed in the XRD analysis. Meanwhile, the reaction between aluminosilicates and alkaline solution was triggered, producing geopolymeric gels, which increased the bonding of the AAM matrix [31,57]. The highest strength was observed in the specimen WZ with NZ, reaching 24.33 MPa, attributed to the unique structure of NZ, which could provide mutual support with hydrates and geopolymeric gels, modifying the pore structure of the AAM by decreasing the microcracks and pores [58] and improving NZ structure creation.

A reduction by 13.28% in the compressive strength was measured after the usage of 20% of CPG in the specimen WP. This differs from the results in the existing investigations using CPG or gypsum in alkali-activated or geopolymer materials, which report an increase in the mechanical strength [20,24,59,60,61]. The reason may be that the dominant precursor WA, which primarily consists of Si and Ca, is used together with Ca-based activator CH as an extra source of active Ca. CH can attract some amount of water available in the binder system, which hinders the CPG hydration and can lead to unreacted CPG particles in the matrix. Along with the existence of CPG, the exceeding Ca supply negatively affects the reaction process. Meanwhile, the reaction of CPG with sodium can produce sodium sulphate (Na_2_SO_4_) crystals, which cover the surface of samples and cause structural defects. This is also pointed out by Zhu [62].

After being attacked by sulphate ambient, for samples W, WF, WM, and WZ, a decrease in the compressive strength was noted, with K between 39.58 and 81.40%. This demonstrates the damage to the mechanical properties by sulphate erosion. One reason could be the leaching of alkaline and calcium ions into the sulphate solutions, which deteriorated the microstructure of the hydrates and increased porosity of the samples [37,46]. In addition, the crystallisation of the sulphate tended to expand in volume, and the precipitates due to the reaction of the sulphate solution and the samples filled the matrix, generating swelling stresses and propagating the development of the cracks [37,63]. The oven-drying process during each cycle in this study, on the other hand, could lead to a rapid loss of physically bonded water from the samples, causing damage to the internal structure. These factors also impacted the volume change in the AAM.

It is noted that the binary addition of CFA, MK, and NZ contributed to an increased resistance to sulphate erosion, i.e., a growth of K from 39.58% for W to 44.49, 39.83, and 81.49%, respectively. This indicates the effects of aluminosilicate precursors to restrain the deterioration of the mechanical properties, ascribed to the co-existence of hydrate and geopolymeric products, providing a compact and interconnected structure as shown in SEM graphs. The generation of a neutralised cross-linked geopolymer structure prevented the ingress of the sulphate solution, determining a better durability against sulphate attacks [53,54]. It is worth mentioning that although WZ exhibited the highest increase in mass and volume, it showed better mechanical properties after sulphate attack, in contrast with WF and WM. This indicates that its changes in mass and volume were mainly associated with the absorption of ambient solution in the NZ chambers, instead of structural damage. Another possibility could be the growing content of sodium ions in the sulphate solutions, which promoted the geopolymerisation reaction [46].

The utilisation of CPG, in contrast, enhanced the compressive strength after sulphate attack by twofold, with a K value of 211.48%. Similar increments in the compressive strength of geopolymer and alkali-activated materials after soaking in sulphate solutions are also reported by other investigations [64,65,66]. This is an indicator of good durability behaviour against the sulphate attacks. Although CPG addition showed negative influences on the 28-day mechanical property development of AAMs, it significantly improved the mortar durability. The reason may be connected to the promotion of CPG hydration in the sulphate ambient, producing a greater quantity of anhydrate hydration products with the increase in sodium sulphate concentration, as confirmed in XRD analysis. This formed a denser microstructure with fewer cracks and pores.

### 3.2. Physical Properties

#### 3.2.1. Visual Appearance

**Figure 5:**
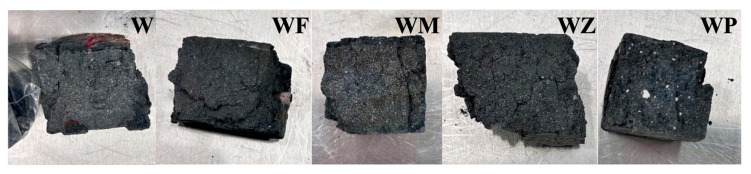
Visual appearance of AAMs subjected to 25 cycles after strength testing.

Figure 5 depicts the macro-morphology of the AAM samples after the sulphate attacks and compressive strength testing. White adherences were observed on the surface of all the samples, which could belong to the crystal precipitates of sodium sulphate (Na_2_SO_4_), as similarly reported by other researchers [36,42]. Its existence was also indicated by the XRD results. This phenomenon was more evident in the samples W and WM, related to a higher degree of degradation. It is noted that in WP, a large white substance was observed, which was unreacted CPG agglomerate owing to the non-homogeneous mixing during the preparation of the sample. In addition, sanding and surface spalling appeared on the samples. This disintegration of the structure enhanced the crack propagation and structural damage when samples were subjected to compression pressure.

#### 3.2.2. Mass Change

**Figure 6:**
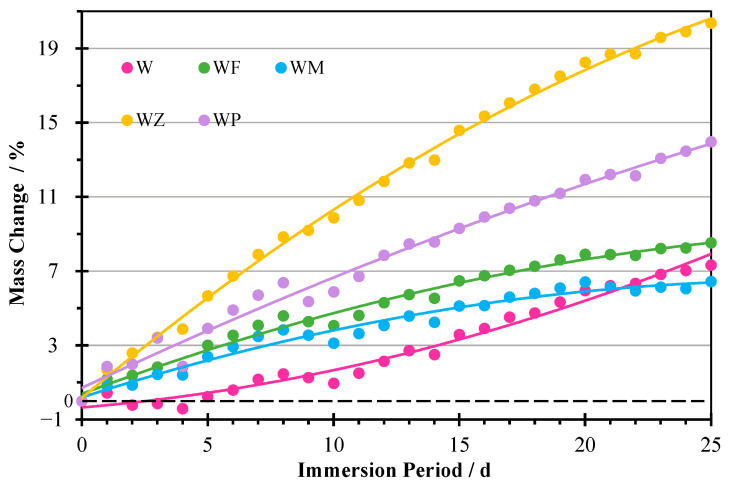
Mass change proportion of AAMs after each cycle.

The mass variations of the AAMs after being subjected to different cycles of sulphate attacks are exhibited in Figure 6. With the increase in immersion time to 25 days, the mass of the tested AAM continued to increase for all five mixtures, within a range of 6.44% to 20.37%. These results align with existing investigations [36,37,42]. The increase in mass is mainly attributed to the absorption of the sulphate solution, which fills the internal pores of the samples [37,67]. A slight decrease in the mass by 0.41% was observed for the specimen W during the first five cycles. This may be associated with the leaching of alkaline ions into the solution in the acidic ambient [40,47]. Another reason may be the damage to the AAM structure, as evidenced in macro-images.

Specimens with NZ (WZ) and CPG (WP) had much greater mass increases than the other samples, reaching 20.37 and 13.96% after 25 cycles, respectively. NZ has a cage-like microstructure made of silica and alumina tetrahedra connected by shared oxygen atoms, which form well-defined channels and chambers inside it [58,68]. This contributes to a web-shaped morphology of WZ as seen in the SEM graph, which can capture and lock water molecules and sulphate ions from the ambient solutions, leading to significant absorption and improvement of the mass. For the specimen WP, it had a loose and incompact microstructure with micropores and cracks, accounting for its remarkable liquid absorption. This is also mentioned by other researchers [69,70]. For the first 20 cycles, the specimen with only WA showed the least mass change. This indicates that the usage of lighter and more porous binary precursor materials enhanced the absorption of the liquid. Meanwhile, it is worth noting that by the end of 25 attack cycles, the mass change of WF, WM, WZ, and WP gradually reached a stability, while the mass increasing rate of W still remained in accumulation. This may be related to the continuous structural deterioration and high LOI of the sample.

#### 3.2.3. Volume Change

**Figure 7:**
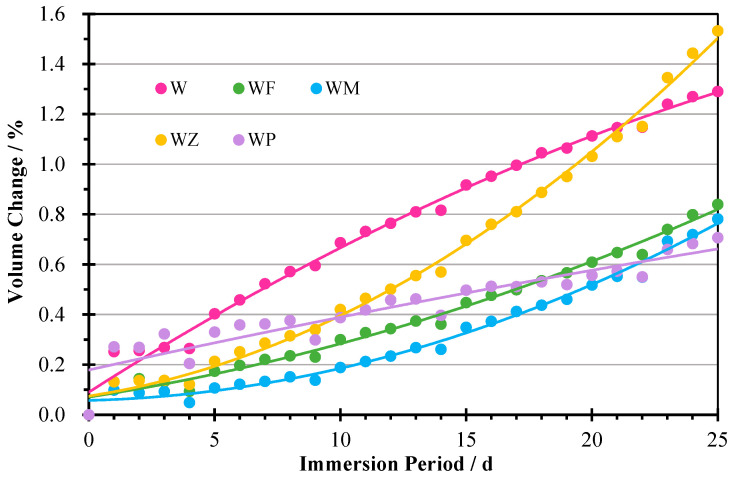
Volume change of AAMs after each cycle.

The volume change of the AAM samples is depicted by the variation in the sample length after exposure to sulphate solution (Figure 7). After 25 cycles, the volume change proportion of the samples varied within the range of 0.71% to 1.53%. For the first 10 days of soaking, the samples expanded slowly, except W with only WA, which exhibited an intensive deformation during the entire 25 cycles. Subsequently, a sharp increase in volume was observed. This indicates that, along with the increasing attack cycles, cracking and damage were inflicted on the samples. Another reason behind the volume expansion was related to the absorption of the sulphate solution filling the micropores of the samples, which introduced internal pressure in the samples [37]. This was also confirmed by the mass increase in the samples (Figure 6).

After 25 cycles of sulphate attack, the volume change rate followed the sequence of WZ > W > WF > WM > WP. The deformation of W was almost two times that of WF, WM, and WP. This indicates that the usage of a binary aluminosilicate or calcium-rich precursor improves the bonding of the mortar matrix and accounts for an increase in the sulphate resistance. It is noted that the specimen WP had a significant increase in mass but the least growth in volume, indicating an increase in its density. This can be due to the existence of spaces between the crystals of CPG, which can capture sodium sulphate from the ambient solutions to its intercrystalline spaces. This is also mentioned by Zhu [62]. As analysed in the XRD outcomes, the exposure to sulphate ambient triggered the hydration reaction of gypsum contents, contributing to a more compact and densified microstructure, which also improved the compressive strength after soaking. This can be noticed in the strength analysis (Figure 4) and SEM graphs. These results imply that the usage of CPG positively improved the sulphate resistance of WA-based AAMs. Ca in the system reacts with sulphate ions, improving the strength due to the densification of the structure. From a long-term perspective, the reaction of calcium and sulphate ions reaches a limitation, and the expansion of the microstructure can propagate the generation of cracks, leading to a loss of mechanical strength. But more investigations are needed to further confirm the effects of sulphate environments on the long-term properties.

Meanwhile, there is no evident correlation between the change in mass and volume. The former is affected by the absorption of ambient solution as well as the deionisation of the hydrates, whereas the latter is determined by the expansion of the pore structure and the production of the erosion products. It is observed that W had a similar volume change after 25 cycles to WZ, but a much smaller mass increase. This is due to the special comb structure of NZ in which the microchambers can absorb and store the ambient solutions, which leads to a great growth in the mass and increasing internal pressure. This is one of the factors influencing its volume expansion besides the generation of the swelling products. Thus, compared to WZ, the expansion products enhanced the volume of W, but it showed a much smaller mass increase.

#### 3.2.4. Relationship Between Mass or Volume Changes and Compressive Strength Loss

**Figure 8:**
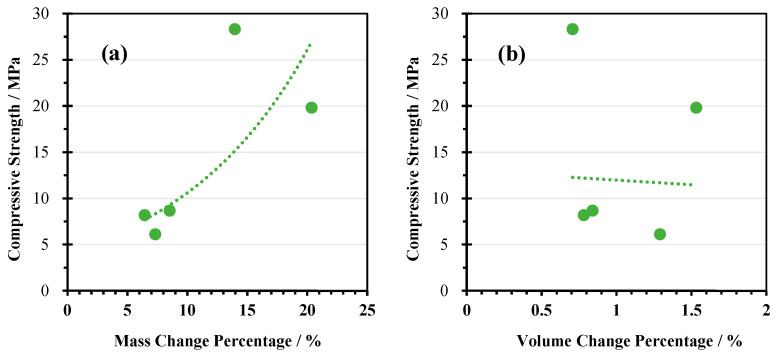
Relationship between compressive strength evolution and (**a**) mass change percentage and (**b**) volume change percentage after sulphate attacks.

In Figure 8, the relationship between the changes in compressive strength along with mass or volume changes after 25 corrosion cycles is presented. In total, a weak positive correlation was seen between compressive strength and mass increase, with a correlation coefficient of 0.74. Meanwhile, no obvious correlation with volume expansion was observed, with a correlation coefficient of −0.09. In total, the sample WP with CPG exhibited the best durability. A significant increase in the strength and mass was noted along with the lowest deformation. For the samples with aluminosilicate-rich precursors, WZ exhibited better durability against sulphate attacks compared to WM and WF.

### 3.3. Microstructural Properties

#### 3.3.1. XRD

**Figure 9:**
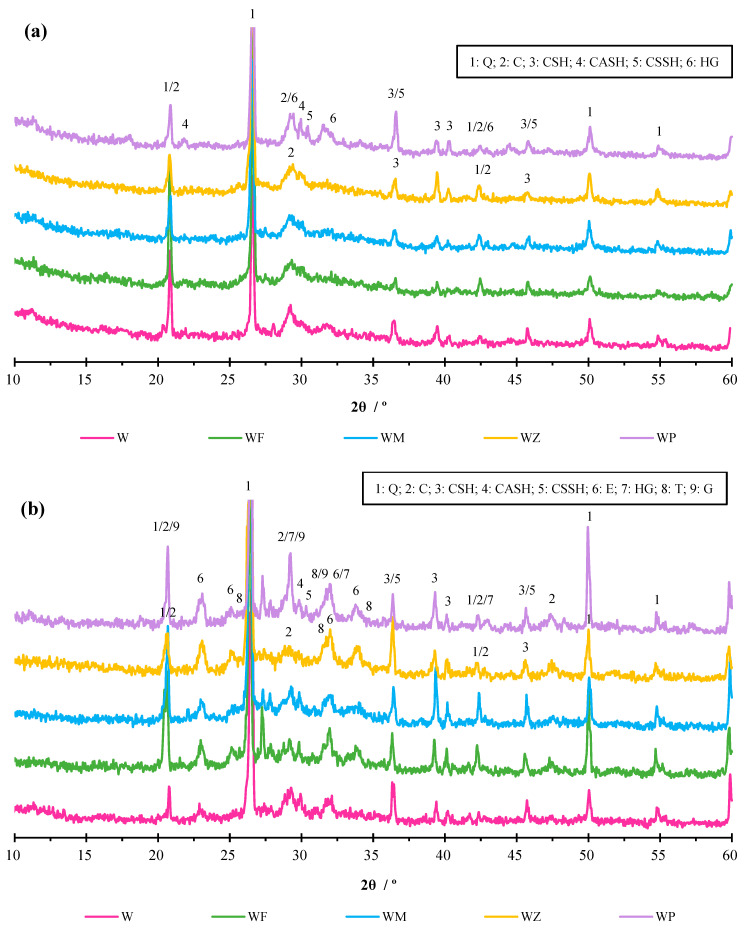
XRD patterns (**a**) before and (**b**) after sulphate attack (Q: quartz, SiO_2_; C: calcite, CaCO_3_; CSH: calcium silicate hydrate; CASH: calcium aluminate silicate hydrate; CSSH: calcium silicate sulphate hydrate; E: ettringite, calcium aluminate sulphate hydrate; HG: hemihydrate gypsum, CaSO_4_·0.5H_2_O; T: thenardite, Na_2_SO_4_; G: gypsum, CaSO_4_·2H_2_O).

The XRD spectra of the AAM samples before and after the sulphate attack are displayed in Figure 9a and Figure 9b, respectively. Before sulphate corrosion, the 28-day XRD pattern showed that the main substances in the produced AAMs encompassed quartz, calcite, calcium silicate hydrate (CSH), and calcium aluminate silicate hydrate (CASH). The last two are major hydration products in Ca-rich alkali-activated materials [71]. Meanwhile, in WP with 20% CPG, a typical sulphate hydrate product, calcium silicate sulphate hydrate (CSSH), was generated in a binder system rich in calcium and sulphate, which is the result of hydrated CPG in an alkaline environment. Also, the peaks of HG were identified, indicating the inhibition of gypsum hydration. The existence of hydration products and an alkaline environment prevents HG hydration from forming well-crystallised gypsum [62], which is also seen in SEM images. Another point worth noting is that a broad hump was detected between 2θ of 25 and 35°, which belonged to the amorphous region of gel hydrates [72], i.e., CSH, CASH, CSSH, and sodium aluminate silicate hydrate (NASH), which is the product of a geopolymerisation reaction between aluminosilicates and alkaline solution. Theoretically, the introduction of aluminosilicate-rich precursors, CFA, MK, and NZ, can trigger geopolymerisation and produce three-dimensional polymeric NASH products, which favours the development of the compressive strength. Due to the poor crystallisation, NASH gel is usually identified as an amorphous hydrate region in XRD patterns, and combined with the results obtained in SEM-EDS, its existence in F20, M20, and Z20 can be confirmed.

After 25 cycles of sulphate attack, in Figure 9b, three new phases were identified, and the intensity of the peaks slightly changed. In all the samples, ettringite (calcium aluminate sulphate hydrate) phase was formed due to the reaction of intrusive sulphate ions with calcium and aluminates in the AAMs. This aligns with the results reported in the existing literature [42,73]. Irbe et al. [74] pointed out that the contents of CH importantly influenced the generation of ettringite phases and promoted the reaction between sulphate and alumina from CASH gels. In the context of this research, WA, as the major precursor material, was rich in Ca, and CH was used as a ternary activator. The availability of large quantities of CH in the system, therefore, facilitated the reaction to form ettringite in the sulphate ambient. Additionally, crystalline phases of thenardite (Na_2_SO_4_) were noted in the XRD patterns, which can be attributed to the precipitation of Na_2_SO_4_ from the sulphate solution absorbed by the AAM samples. The discovery of these two phases agrees with the findings of the SEM-EDS results and macrostructural analysis. In the sample WP with 20% CPG, besides ettringite and thenardite, gypsum (CaSO_4_·2H_2_O) was generated, which was the result of further hydration of anhydrate and hemihydrate gypsum, and the reaction between sulphate and calcium ions in a sulphate- and calcium-rich system. The production of new erosion phases, ettringite and gypsum, is related to the propagation of degradation cracking in the AAMs and results in volume expansion, which is confirmed in SEM graphs and the volume change behaviour of the samples. Another point worth noting is the reduced amorphous region, indicating the decomposition of hydrate gels in the acidic ambient, which negatively impacted the densification of the microstructure and contributed to the strength loss. Notwithstanding, strength development was measured in WP after 25 cycles. The reason may be the promotion of anhydrate hydration in a sulphate-rich environment that facilitated the production of phases containing calcium and sulphate, as evidenced in the XRD patterns where the peaks belonging to CSSH, hemihydrate, ettringite, and gypsum showed increasing intensities.

#### 3.3.2. SEM-EDS

**Figure 10:**
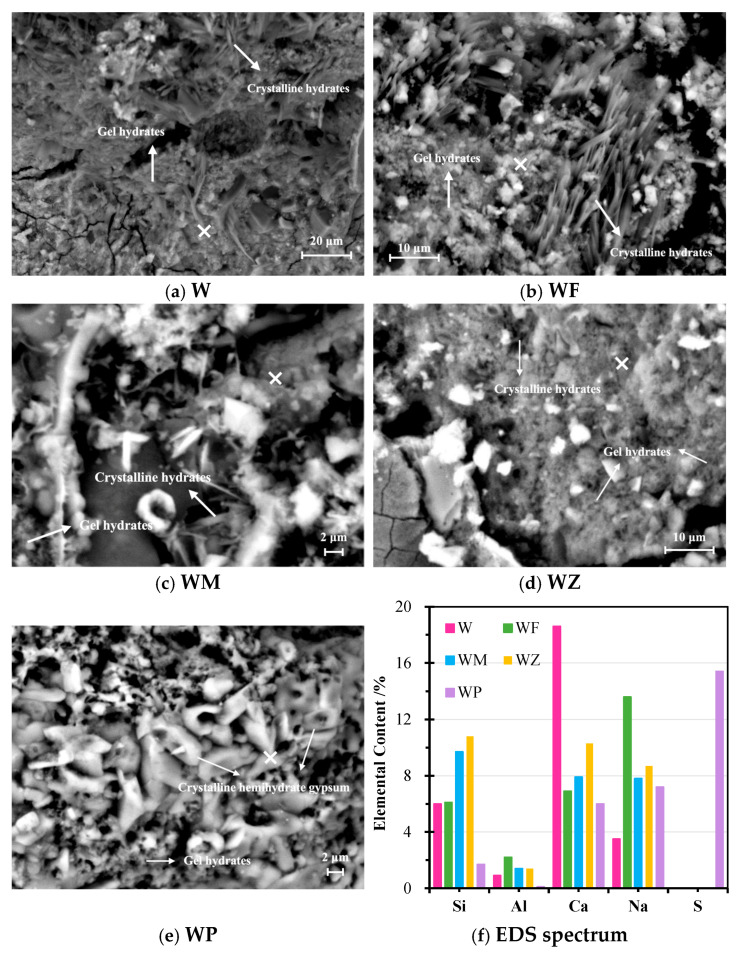
SEM images and EDS spectrum of AAMs before sulphate attack (×: testing point of EDS).

**Figure 11:**
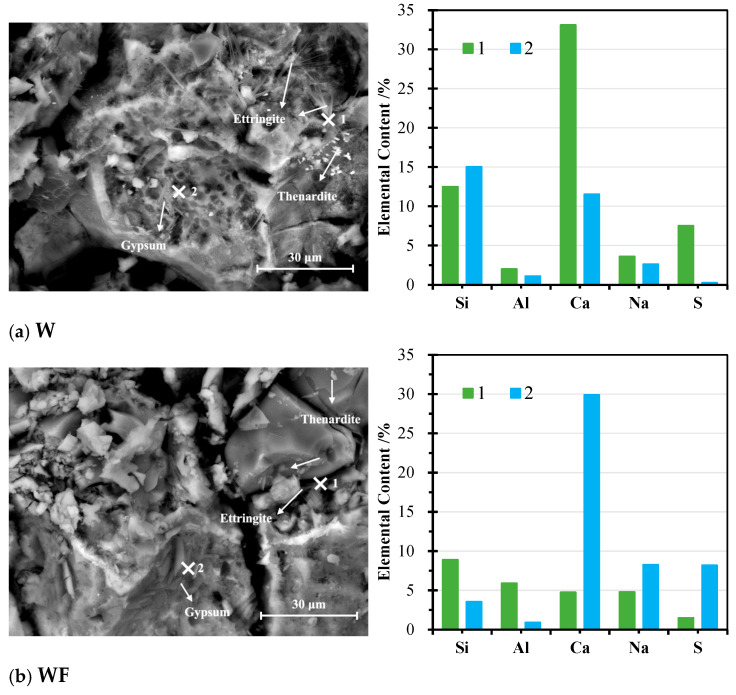

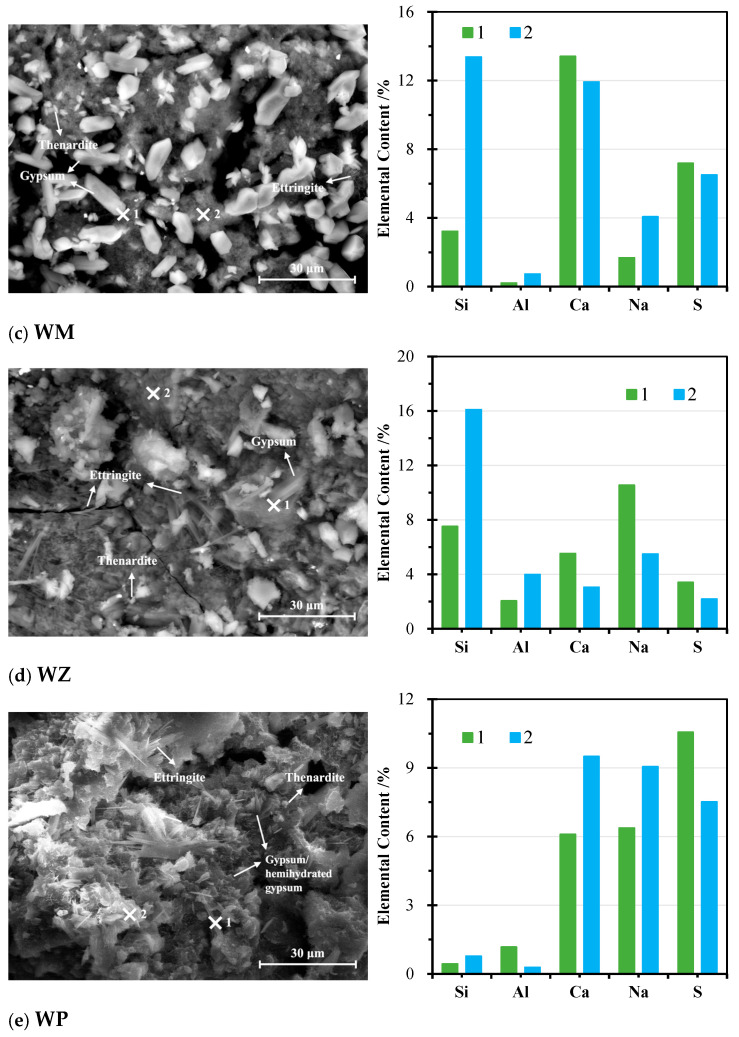
SEM images and EDS spectrum of AAMs after sulphate attack (×: testing point of EDS).

In Figure 10 and Figure 11, micromorphology and elemental contents of AAMs before and after sulphate corrosion are compared. Well-crystallised hydrate products in the shape of needles were observed in the sample W with only WA before sulphate corrosion in Figure 10a. The generation of gel hydrates contributed to a well-connected matrix structure. These agreed with the results of XRD. However, there was a large number of microcracks and pores, which limited the strength development of the sample. In contrast, the incorporation of binary aluminosilicate precursors, seen in the samples WF, WM, and WZ, densified the microstructure of AAMs. Particularly in the sample WZ with 20% of NZ where the highest amount of Si is detected, a compact web-shaped structure was detected. Unreacted NZ particles and gel hydrates, rich with active Si, formed a composite structure, increasing the internal bonding. This interaction can improve the pore structure by providing a multi-scaled pore distribution with micropores of NZ and mesopores and macropores of gel products [58,68,75]. This greatly favours the development of strength, as discussed in the mechanical properties. It is worth noting that the existence of both gel and crystalline hydrates significantly compacts the microstructure of AAMs, with crystalline hydrates acting as microfillers that bridge the cracks and gaps, and with gel hydrates as strong supports. With 20% CPG, in Figure 10e, WP displayed a mal-bond and nonhomogeneous morphology, creating a very porous microstructure. The rod-shaped crystals were observed, which belonged to the fractured hemihydrated gypsum plates. Their existence introduced pores and gaps into the binder matrix, deteriorating the mechanical property development. The alkaline ambient negatively affected gypsum hydration and crystallisation, as also mentioned by Zhu et al. [62]. Although gel-shaped hydrates were identified in the morphology, they failed to connect the hemihydrated gypsum crystals effectively.

The EDS results detected in the area of gel products are exhibited in Figure 10f, together with the data summarised in Table 5. Based on the elemental contents of the hydrates, the sample W was dominated by Ca-based hydrates, particularly CSH. In the samples WF, WM, and WZ, the ratios of Na and Al greatly increased, indicating the formation of a growing content of CASH and NASH. The latter belongs to the geopolymeric product. Its identification testifies to the effects of aluminosilicate-rich precursors, which trigger geopolymerisation and lead to the improvement of compressive strength [57,76]. The specimen WP was mainly composed of S, Ca, and Na, and no Al was detected, which agrees with the identification of hemihydrate gypsum and CSSH in XRD. There is also the possibility of Na_2_SO_4_ formation, which, however, was not detected in XRD. The reason for this may be its high dissolution and dilution in the binder system, affecting the identification of its crystals [42].

Figure 11 shows the morphology of AAMs after 25 cycles of exposure to sulphate solution, as well as the corresponding EDS spectra, which were also summarised in Table 6. In comparison to the microstructure before sulphate attacks, thenardite crystals were distributed in the matrix, which aligns well with the results in XRD analysis and macro-appearance. The temperature treatment at 103° in each cycle promoted the efflorescence of Na_2_SO_4_ from the solution. A propagation of microcracks and voids was observed in the sample W in Figure 11a, which presented a honeycomb-shaped structure. This resulted from the swelling damage in the matrix due to the decomposition of hydrates under sulphate exposure and the production of expansive corrosion products. This observation explains the great loss in compressive strength and is consistent with the existing findings [36,77]. Needle-like clusters were detected in the morphology, and combined with their EDS results at point 1, which indicated the primary contents of Si, Ca, S, and Al, it is suggested that the crystals can be ettringite. The elemental composition of gel hydrates at point 2 showed a sharp decrease in the Ca level, demonstrating that Ca-based hydrates, CASH and CSH, underwent a decalcification, which accounted for the skeleton deterioration of the hydrates [73,78].

Similarly, in WF with 20% of CFA in Figure 10b, large visible gaps and voids existed in the morphology. This indicates that the binary usage of CFA has a limited effect on improving the durability of WA-based AAMs against sulphate attack. The EDS outcomes of the needle-shaped substances at point 1 suggested the existence of ettringite, and the slab-like crystals overpacked together, which were composed primarily of Ca and S at point 2, belonged to gypsum. A very porous microstructure with a loose matrix was seen in the sample WM in Figure 11c. A great deterioration in the structure is responsible for the great strength loss after sulphate attack. A large number of rod-like crystals were distributed on the surface of the matrix in WM, which had a primary elemental composition of S and Ca (point 1) and belonged to gypsum. Based on the EDS results of the matrix at point 2, gel hydrates co-existed with ettringite, which showed a rosette-like structure when the crystals agglomerated together. The formation of expansive gypsum and ettringite, filling the gaps between crystals, improved the internal stress, which is one of the reasons for crack propagation [79]. A compact microstructure was shown in the sample WZ with 20% NZ in Figure 11d, suggesting a better resistance against sulphate attack. This result agrees with the strength development. Erosion products, ettringite and gypsum, were identified. But the matrix structure remained intact, suggesting a well-established microstructure formed of hydrates and unreacted NZ. In the sample WP with 20% CPG in Figure 11e, well-crystallised hemihydrate gypsum and gypsum plates (seen in EDS results of point (1) were densely packed together, with ettringite clusters (EDS results of point (2) growing in the voids. Compared to the morphology of WP before sulphate immersion, a more compact and homogeneous microstructure was observed. This indicates that curing in Na_2_SO_4_ solution promoted anhydrate hydration, favouring structural development. This finding is consistent with XRD and strength tests.

## 4. Conclusions

This study investigated the sulphate resistance of WA-based AAMs with CFA, MK, NZ, and CPG as binary precursors at 20% by total precursor mass. The samples were exposed to 25 cycles of 14% Na_2_SO_4_·10H_2_O solution. After being subjected to the sulphate attack cycles, hydrates underwent decalcification and dealumination, releasing Ca and Al ions to react with sulphate and producing swelling erosion products, ettringite and gypsum, which caused volume expansion and microstructural defects.

The reference sample W with only WA showed a homogeneous microstructure composed mainly of CSH-type hydrates. The high carbon content in the WA negatively affected its reaction degree in the activation process. This restrained strength development and durability against sulphate attacks. The immersion in the sulphate cycles contributed to the strength loss coefficient of less than 40%.

The usage of 20% aluminosilicate-based binary precursors CFA, MK, and NZ was associated with a compressive strength increase of 26.15, 33.02, and 57.58%, respectively. The growing content of Al in the system facilitated the production of CASH type hydrates, and the geopolymerisation reaction between aluminosilicates and activators generated polymeric NASH gel hydrates. The co-existence of different types of hydrates improved the microstructure and enhanced the sulphate resistance. This improved the strength loss coefficient up to 81.40%. Compared to CFA- and MK-incorporated samples, a better durability was seen in the sample WZ with NZ. A well-bonded matrix structure supported by hydrates and unreacted NZ, acting as a skeleton, reduced the erosion damages created by ettringite and gypsum generation.

The introduction of 20% Ca-rich binary precursor CPG, due to possible thenardite formation, decreased the mechanical properties of the AMM sample by 13.28% but significantly improved the sulphate resistance. The soaking of the sample in Na_2_SO_4_ solution provided an extra source of sulphate, facilitating anhydrate hydration, generating more CSSH gels and hemihydrate gypsum. The absorption of sulphate solution and the production of new ettringite and gypsum phases increased the mass and volume of the sample and meanwhile contributed to a more homogeneous and compact microstructure, which improved the compressive strength by twofold.

Although the produced materials exhibited good durability against sulphate attacks in an extreme condition, long-term durability tests for one or two years are suggested for future research, together with other categories of durability testing, such as chloride resistance and carbonisation. Considering the strength evolution of the samples with CPG, it is suggested, for further research, to investigate the effects of sulphate environments or water curing on CPG-based materials.

## Figures and Tables

**Table 1:** Chemical composition of raw materials (%).

Material	SiO_2_	Al_2_O_3_	CaO	Fe_2_O_3_	MgO	Na_2_O	K_2_O	P_2_O_5_	SO_3_	LOI
WBA	50.26	9.20	19.99	1.90	6.44	1.19	5.71	3.34	0.51	2.73
WFA	22.91	2.63	31.50	2.33	3.57	0.26	4.01	2.71	0.88	26.93
CFA	37.00	22.80	1.92	3.63	0.88	0.50	2.96	0.73	0.18	5.20
MK	54.40	31.90	2.82	0.42	0.44	5.46	0.50	0.13	0.13	1.10
NZ	71.5	13.1	3.22	1.66	0.81	0.60	2.99	0.03	0.01	-
CPG	1.95	0.69	53.10	0.09	-	0.21	0.03	0.79	36.70	6.70

**Table 2:** Mix design for 1 m^3^ of mortar (kg).

Mix	WBA	WFA	CFA	MK	NZ	CPG	RS	SH Solution	WG	CH	BA
W	273.44	273.44	-	-	-	-	1093.75	300.82	168.36	54.69	5.47
WF	218.75	218.75	109.38	-	-	-	1093.75	300.82	168.36	54.69	5.47
WM	218.75	218.75	-	109.38	-	-	1093.75	300.82	168.36	54.69	5.47
WZ	218.75	218.75	-	-	109.38	-	1093.75	300.82	168.36	54.69	5.47
WP	218.75	218.75	-	-	-	109.38	1093.75	300.82	168.36	54.69	5.47

**Table 3:** Mix ratio of the produced mortar (% by the total precursor mass).

Mix	WBA	WFA	CFA	MK	NZ	CPG	RS	SH Solution	WG	CH	BA
W	40	40	-	-	-	-	200	55	31	10	1
WF	40	40	20	-	-	-	200	55	31	10	1
WM	40	40	-	20	-	-	200	55	31	10	1
WZ	40	40	-	-	20	-	200	55	31	10	1
WP	40	40	-	-	-	20	200	55	31	10	1

**Table 4:** Summary of compressive strength change.

Compressive Strength	W	WF	WM	WZ	WP
Before sulphate attack/MPa	15.44	19.48	20.54	24.33	13.39
After sulphate attack/MPa	6.11	8.67	8.18	19.81	28.32
Strength loss coefficient/%	39.58	44.49	39.83	81.40	211.48

**Table 5:** EDS data of the AAM samples before sulphate attack (%).

Sample	C	O	Si	Al	Ca	Na	K	S
W	14.8	46.5	6	0.9	18.6	3.5	3.9	0
WF	16.9	51.2	6.1	2.2	6.9	13.6	1.6	0
WM	12.2	55.1	9.7	1.4	7.9	7.8	2.3	0
WZ	16	49.6	10.8	1.4	10.3	8.7	2.5	0
WP	7.8	35.4	1.7	0.1	6	7.2	25.5	15.4

**Table 6:** EDS data of the AAM samples after sulphate attack (%).

Sample	C	O	Si	Al	Ca	Na	K	S
W	7.71	30.43	12.51	2.01	33.11	3.61	3.1	7.52
WF	7.91	35.05	3.53	0.91	29.9	8.26	6.04	8.18
WM	21.7	52.21	3.22	0.21	13.41	1.68	0.39	7.18
WZ	15.24	54.17	7.52	2.05	5.53	10.55	1.53	3.41
WP	17.63	55.37	0.43	1.17	6.1	6.37	1.57	10.56

## Data Availability

The original contributions presented in this study are included in the article. Further inquiries can be directed to the corresponding author.
